# Acute thrombosis of the abdominal aorta in a patient with inoperable lung cancer

**DOI:** 10.1186/1749-8090-8-S1-P117

**Published:** 2013-09-11

**Authors:** K Ergunes, H Iner, I Yurekli, H Cakır, L Yilik, A Gurbuz

**Affiliations:** 1 Izmir Katip Celebi University Ataturk Training and Research Hospital, Department of Cardiovascular Surgery, Izmir, Turkey

## Background

Acute thrombosis of the abdominal aorta in patients with cancer is excessively rare but potentially devastating complication. We report a case of acute thrombosis of the abdominal aorta in patient with inoperable lung cancer.

## Methods

A 68-year old male with lung cancer was admitted to our hospital. He had paraplegia of lower extremities. His both femoral pulses were non palpable, cold, and cyanotic. The Doppler USG examination was unable to detect any pulse at the bilateral common femoral arteries. Contrasted computed tomographic angiography revealed occlusion of abdominal aorta and both iliac arteries. Transthoracic echocardiography revealed no atrial thrombus. He was diabetic and his leukocyt count was 21.08 K/uL.

## Results

Both femoral arteries were explored. Thrombectomy was applied abdominal aorta, iliac arteries, and both lower extremity arteries using a balloon-tip Fogarty catheter. Large amount of fresh thrombus material was retrieved from the aorta and right external iliac artery. Left external iliac artery was atherosclerotic and stenotic. A femoro-femoral 8 mm PTFE graft bypass was performed between right and left common femoral arteries. After operation, both lower extremity distal pulses were detected with the Doppler USG. This patient died on fourth postoperative days due to respiratory and renal failure.

## Conclusions

Acute abdominal aortic occlusion in patients with lung cancer is a catastrophic condition. High morbidity and mortality rate occur even treated in a timely manner.

